# Ultra-processed foods: a fit-for-purpose concept for nutrition policy activities to tackle unhealthy and unsustainable diets

**DOI:** 10.1017/S1368980022002117

**Published:** 2023-07

**Authors:** Mark Lawrence

**Affiliations:** Institute for Physical Activity and Nutrition (IPAN), Deakin University, Geelong, Australia

## Abstract

Modern nutrition science began approximately 100 years ago in the context of nutrient deficiency diseases. Nutrition research and policy activities were framed mostly within a reductionist paradigm in which foods were analysed as being a collection of their constituent nutrients. Today, nutrition problems extend to all forms of malnutrition as well as environmental sustainability considerations and are associated with food and dietary pattern exposures. In 2009, researchers investigating the nutrition transition in Brazil proposed that industrial food processing was a key determinant of nutrition problems. The NOVA food classification system which is based on the nature, extent and purposes of food processing was developed to operationalise this proposition. The ultra-processed food (UPF) concept within NOVA is receiving much attention in relation to nutrition research and policy activities. This commentary describes the UPF concept as being fit-for-purpose in providing guidance to inform policy activities to tackle unhealthy and unsustainable diets. There is now a substantial body of evidence linking UPF exposure with adverse population and planetary health outcomes. The UPF concept is increasingly being used in the development of food-based dietary guidelines and nutrition policy actions. It challenges many conventional nutrition research and policy activities as well as the political economy of the industrial food system. Inevitably, there are politicised debates associated with UPF and it is apparent a disproportionate number of articles claiming the concept is controversial originate from a small number of researchers with declared associations with UPF manufacturers. Prominent examples of these claims are assessed.

## The nutrition policy challenge

Modern nutrition science began approximately 100 years ago within the context of nutrient deficiency diseases^(1)^. A ‘Nutrient Deficiency’ era of nutrition science prevailed over the following decades^(2)^. During this nascent period, the nature and scope of nutrition science were framed mostly within a reductionist paradigm in which foods and dietary patterns were analysed as being a collection of their constituent nutrient parts. Nutrition research activities focused on studying associations between single nutrients and specific diseases and isolating and synthesising vitamins and minerals. These research activities informed the development of nutrition policy activities including the first nutrient reference values and food fortification interventions.

Over the following century, there was a series of social, political, economic and technological changes affecting the structure and operation of global food systems which in turn have affected food supplies, dietary behaviours and nutritional health outcomes. The Nutrient Deficiency era gave way to the ‘Dietary excess and imbalances’ era and most recently the ‘Food System Sustainability’ era^(2)^. Nutrition science has increasingly been framed within a holistic paradigm in which foods and dietary patterns are analysed as being more than the sum of the nutrients they contain^(3)^. Nutrition studies are reporting these food and dietary pattern exposures are associated with contemporary nutrition problems. Yet, the design of many nutrition policy activities continues to be informed by a nutrition science approach operating within a reductionist paradigm better suited to addressing nutrition problems prevalent a century ago. Nutrition policymakers are being challenged to apply a more fit-for-purpose conceptual basis to the design of nutrition policy activities tackling unhealthy and unsustainable diets^(1,2,4)^.

## An innovative concept to address this challenge

During the early 2000s, Carlos Monteiro and his research team at the USP centre (Center for Epidemiological Studies in Health and Nutrition), University of São Paulo began investigating the nutrition transition in Brazil. Analysing trends in the data recorded in a series of national household budget surveys, they noted that Brazilian households were reducing their purchasing of staple foods such as beans, rice and vegetables as well as culinary ingredients and replacing them with ready to consume products, such as salty snacks, soft drinks and ready meals. They also observed that these food purchasing trends coincided with increasing prevalence in obesity and diet-related chronic diseases among the Brazilian population.

Drawing on the findings from his team’s investigation into the Brazilian nutrition transition, in 2009 Monteiro published a commentary in *Public Health Nutrition* which presented a new theory for predicting and explaining relationships between food and health^(5)^. The theory proposed that industrial food processing was a core influence on the structure and operation of food systems around the world and consequently a key determinant of dietary patterns and diet-related health outcomes. Critically, to operationalise the theory he introduced the innovative NOVA food classification system which is based on the nature, extent and purposes of food processing. NOVA classifies foods into four groups: group 1 – unprocessed and minimally processed foods; group 2 – processed culinary ingredients; group 3 – processed foods and group 4 – ultra-processed foods (UPF). It is the concept of UPF (Group 4) which has become the most germane component of the NOVA food system in its application to nutrition research and policy activities. UPF are defined as ‘*formulations of ingredients, mostly of exclusive industrial use, that result from a series of industrial processes*’^(6)^. Examples of such products include, margarines, soft drinks, preprepared frozen meals, instant noodles and confectionery.

In a letter which the journal had invited me to submit in response to Monteiro’s commentary I supported the proposed theory and briefly mentioned it also had relevance to sustainability considerations in the context of the association between energy use and degree of food processing and packaging^(7)^. Monteiro concurred saying ‘this is another reason to avoid ultra-processed foods’^(8)^, p. 1968. Since 2009, recognition of the UPF concept’s relevance to sustainable diets has grown substantially^(9,10)^.

As the twenty-first century unfolds, a rapid proliferation in the manufacture and consumption of UPF globally, regionally and nationally is being observed^(11)^. Although the volumes and variety of UPF are highest in higher income countries, where it is estimated they now contribute more than half of the population’s dietary energy intake^(12)^, the rate of change in consumption is especially dramatic in highly populated middle-income countries. These findings are linked with the industrialisation of food systems and consistent with the proposition that UPF are a powerful marker of the global nutrition transition^(11)^.

## Evidence supporting the concept

There is now a substantial body of evidence linking UPF exposure with adverse population and planetary health outcomes. The evidence has been collected from observational, experimental and mechanistic studies conducted in a diversity of countries. A recent systematic review and meta-analysis reported that UPF consumption was associated with increased risk of obesity, all-cause mortality, the metabolic syndrome, depression, cardiometabolic diseases and frailty among many other chronic diseases in adults as well as the metabolic syndrome in adolescents and dyslipidaemia in children^(13)^. Importantly, the observed associations between UPF and these diseases are not just the result of poor nutrient profiles. A randomised controlled trial conducted by Hall and colleagues showed that consuming an ultra-processed diet matched for macro-and micro-nutrient composition with the control diet caused a significant increase in *ad libitum* energy intake and consequent weight and body fat gain^(14)^. Evidence from mechanistic studies is lending biological plausibility to the observed associations by showing how the novel chemical compositions and/or food matrix structures of UPF might be acting through one or more physiological, immunological, hormonal or neurobiological pathways^(15,16)^.

In relation to dietary sustainability metrics, a review which summarised the magnitude and types of environmental impacts resulting from each stage of the UPF supply chain reported three core findings^(17)^: (i) UPF production uses significant finite environmental resources; (ii) UPF are responsible for significant environmental degradation and waste; and (iii) findings (i) and (ii) are all the more egregious from a sustainability perspective when it is considered that UPF are superfluous to basic human needs.

There are three characteristics of the body of evidence linking UPF exposure with adverse population and planetary health outcomes which have particular salience for the utility of the concept in guiding the formulation of nutrition policy activities:

- The risk exposure is a combination of increased dietary intake of UPF and reduced dietary intake of NOVA groups 1 and 3 (displaced by UPF).
- UPF refers to a heterogeneous group of products in relation to their health and sustainability effects, and it is the overall dietary pattern (amounts and combinations of individual UPF) rather than the intake of individual UPF which is relevant to explaining and predicting health and sustainability outcomes^(18)^.
- Humans have not evolved with the ability to efficiently metabolise the novel chemical compositions and physical structures of many UPF^(4)^.

## The utility of ultra-processed foods as a fit-for-purpose concept for nutrition policy activities

Nutrition policymakers are increasingly embracing the UPF concept for its fit-for-purpose guidance in formulating nutrition policy activities to tackle unhealthy and unsustainable diets. Recommendations to avoid or reduce UPF consumption have been incorporated into national dietary guidelines published in Brazil, Uruguay, Peru, Ecuador, Israel and Malaysia^(19)^ and in France a target was set to reduce UPF consumption by 20 % between 2018 and 2021^(20)^. Similarly, dietary guidance from the American Heart Association recommends avoidance of UPF^(21)^. In a follow-up to the 2021 United Nations Food Systems Summit, the ‘Workplan of the Coalition of Action for Healthy Diets from Sustainable Food Systems for Children & All’^(22)^ has recommended avoidance of UPF in its special project on food-based dietary guidelines incorporating sustainability.

A special report on UPF published by the UN Food and Agriculture Organization^(12)^ as well as a number of expert commentaries^(19,23,24)^ have drawn attention to the need for nutrition policy actions to support the implementation of UPF-related dietary guideline recommendations. Commonly identified policy actions to help reduce the consumption of UPF include front-of-pack labelling information, taxes, media campaigns and marketing restrictions. Conversely, policy actions directed towards amending the decision-making processes which facilitate the entry of UPF into the food supply receive limited attention. For example, reforming the nature and scope of the risk assessment process used in the setting of food standards which relate to the preparation and marketing of UPF. The UPF concept is yet to be formally recognised within the risk assessment activities of the Codex Alimentarius Commission and national food standards agencies. Several researchers are now calling for risk assessment procedures in the setting of food standards associated with UPF to extend from their current focus on food safety to also address broader social, ecological and public health considerations^(25,26)^.

## The politicised nature of the scientific debate with the ultra-processed food concept

Nutrition science has a history of competing worldviews over how nutrition problems and solutions are framed and scientific methods and metrics are selected and applied. Healthy scientific debate is a strength of nutrition science as it contributes to the refining and strengthening of nutrition ideas, research and policy practice. Indeed, the outcomes from robust debates have contributed to revisions of nutrient reference values and dietary guidelines, and adjustments to the UPF concept since 2009 are consistent with this dynamic process.

Scientific debate associated with the UPF concept is particularly understandable because it is operationalising a theory which challenges the reductionist paradigm currently dominating many nutrition science activities. Also, policy activities consistent with the UPF concept seek to transform the ultra-processed profile of contemporary food supplies and dietary patterns to a profile consisting mostly of NOVA groups 1–3. This transformation represents a fundamental challenge to the political economy of the industrial food system.

As research and policy attention towards UPF have increased, it has become apparent that a disproportionate number of articles claiming the concept is controversial originate from a small number of researchers with declared associations with UPF manufacturers. Many of these researchers cross reference each other’s similar claims which then are amplified by industry lobby groups, for example in reports used in engagements with nutrition policy events such as the 2021 UN Food Systems Summit^(27)^. Frustratingly, the scientific debate associated with the UPF concept has become highly politicised and the integrity of the claims presented by researchers with UPF associations demands close scrutiny.

Claims raised by researchers with declared associations with UPF manufacturers broadly fit into one or other of three types. First, claims criticising the conceptual basis to NOVA. For example, several researchers question NOVA’s robustness because of observed misalignments between the system’s food classifications and those of pre-existing nutrient profiling models^(28,29)^. These claims are based on a misunderstanding of the conceptual rationale underpinning NOVA. NOVA explicitly seeks to operationalise a holistic (food/dietary pattern) paradigm of nutrition science as distinct from a conventional reductionist (nutrient) paradigm. In this context, misalignments are logically predictable and an alternative explanation for their occurrence may be inherent conceptual limitations with pre-existing nutrient profiling models as tools for informing policy activities to tackle contemporary nutrition problems.

Second, claims about technical aspects of UPF. For example, some researchers have published survey data which they claim show the UPF concept is poorly defined and vulnerable to high inter-rater variability^(30)^. These data were collected from a survey which involved a convenience sample of ‘evaluators’ untrained in NOVA criteria. By contrast, the findings of a study the authors of which had no financial or non-financial competing interests to declare showed when NOVA was evaluated with trained individuals there was less than 5 % disagreement in assessments among those individuals^(31)^.

Third, claims about the application of the UPF concept. For example, a researcher associated with grain industries states that all foods with added nutrients are UPF and consequently this broad-based classification risks adversely affecting dietary quality and hindering certain public health food fortification interventions^(32)^. However, this claim is factually incorrect as NOVA group 1 foods include ‘foods with vitamins and minerals added generally to replace nutrients lost during processing, such as wheat or corn flour fortified with iron and folic acid’^(12)^ (page 11). In another example, researchers associated with soyabean-related industries claim that classifying alternative plant protein foods as UPF risks lowering their public acceptance and stifling incentives for food processing innovations to help promote public health and reduce the environmental footprint of diets^(33)^. Missing from such claims is a critical comparison of the broader public health, environmental and social implications of such innovations relative to food processing innovations to promote existing non-UPF nutritious plant-source protein foods such as minimally processed legumes and nuts.

## Future ultra-processed food research priorities

UPF is a fit-for-purpose concept for guiding nutrition policy activities to tackle unhealthy and unsustainable diets. In future, the concept’s utility will likely be further strengthened by ongoing research activities to build the body of evidence of associations between UPF and adverse population and planetary health outcomes. In particular, more epidemiological research is needed to investigate the impact of UPF intake on all forms of malnutrition in infants, children and adolescents in all regions of the world. More mechanistic studies of the impact of UPF on physiological, immunological, hormonal and neurobiological pathways are needed to support the interpretation of the epidemiological evidence. Systematic research is also required to investigate the impact of UPF production, distribution, consumption and waste on sustainability metrics with a particular need for evidence of impact on biodiversity, eutrophication, soil health and atmospheric aerosol pollution^(17)^. The ability to conduct this research will be increased by investing in surveys to collect data on the type and timing of UPF entering the marketplace and their sales as well as UPF consumption^(34,35)^.
